# Discovery of Novel Allosteric Effectors Based on the Predicted Allosteric Sites for *Escherichia coli* D-3-Phosphoglycerate Dehydrogenase

**DOI:** 10.1371/journal.pone.0094829

**Published:** 2014-04-14

**Authors:** Qian Wang, Yifei Qi, Ning Yin, Luhua Lai

**Affiliations:** 1 BNLMS, State Key Laboratory for Structural Chemistry of Unstable and Stable Species, College of Chemistry and Molecular Engineering, Peking University, Beijing, China; 2 Center for Quantitative Biology, Peking University, Beijing, China; University of Bologna & Italian Institute of Technology, Italy

## Abstract

D-3-phosphoglycerate dehydrogenase (PGDH) from *Escherichia coli* catalyzes the first critical step in serine biosynthesis, and can be allosterically inhibited by serine. In a previous study, we developed a computational method for allosteric site prediction using a coarse-grained two-state Gō Model and perturbation. Two potential allosteric sites were predicted for *E. coli* PGDH, one close to the active site and the nucleotide binding site (Site I) and the other near the regulatory domain (Site II). In the present study, we discovered allosteric inhibitors and activators based on site I, using a high-throughput virtual screen, and followed by using surface plasmon resonance (SPR) to eliminate false positives. Compounds 1 and 2 demonstrated a low-concentration activation and high-concentration inhibition phenomenon, with IC_50_ values of 34.8 and 58.0 µM in enzymatic bioassays, respectively, comparable to that of the endogenous allosteric effector, L-serine. For its activation activity, compound 2 exhibited an AC_50_ value of 34.7 nM. The novel allosteric site discovered in PGDH was L-serine- and substrate-independent. Enzyme kinetics studies showed that these compounds influenced K_m_, k_cat_, and k_cat_/K_m_. We have also performed structure-activity relationship studies to discover high potency allosteric effectors. Compound 2-2, an analog of compound 2, showed the best *in vitro* activity with an IC_50_ of 22.3 µM. Compounds targeting this site can be used as new chemical probes to study metabolic regulation in *E. coli.* Our study not only identified a novel allosteric site and effectors for PGDH, but also provided a general strategy for designing new regulators for metabolic enzymes.

## Introduction

D-3-phosphoglycerate dehydrogenase (PGDH, EC 1.1.1.95) catalyzes the first critical step in *de novo* L-serine biosynthesis, facilitating the transition of 3-phosphoglycerate (3-PG) into 3-phosphohydroxypyruvate (pPYR) with NAD^+^ as a cofactor [1]. It can be allosterically inhibited by serine, the end product of the pathway [2].

PGDH forms a tetramer composed of four identical subunits, each of which contains three distinctive domains: the substrate-binding domain, the nucleotide-binding domain, and the regulatory domain [3]. The enzyme can be described as a dimer of dimers [3]. Two fundamental dimers, each formed by a contact of the nucleotide-binding domain, further dimerize through contacts of the regulatory domains. L-serine binds to the two adjacent regulatory domains forming a hydrogen bond network [2]. It has been suggested that the binding of serine stabilizes the regulatory domain interface contacts and inhibits enzyme activity by limiting the movement of the rigid domains through flexible hinges, thus preventing the active sites from closing [4].

PGDH undergoes V-type allosteric regulation in which the binding of the effector, L-serine, primarily affects the maximal reaction rate of the enzyme rather than the binding affinity of substrate [2], [5]. Previous studies have shown that both the active site and the serine binding site exhibit a type of half-site activity, i.e., maximal reaction rate or inhibition of catalytic activity can be reached when only two of the four sites are occupied [4], [6].

Up to now, PGDH has only been studied extensively in a few organisms, like *E. coli*, and those studies mainly focused on catalytic and physiologically allosteric mechanisms. In general, all PGDHs from bacterial species are sensitive to L-serine [7]–[10], while those of mammalian species are not [11]–[13], since critical amino acid residues for L-serine allosteric regulation appear to be missing in mammalian PGDHs. Although L-serine is available from dietary sources, it plays a critical role in the synthesis of amino acids, neuromodulators, phosphatidylserine, sphingolipids, purines, and porphyrins. It has also been found that the gene encoding PHGDH (D-3-phosphoglycerate dehydrogenase from human), which controls flux from glycolysis into the serine biosynthesis pathway, is a possible oncogene [14]–[17] and PHGDH amplification is most commonly found in human melanoma [18]. Previous studies in 150 human melanoma samples showed that 39% of the samples exhibited PHGDH copy number gain and high protein expression [17], [18]. Furthermore, disordered L-serine biosynthesis resulting from PHGDH deficiency in children is characterized by congenital microcephaly, psychomotor retardation, and seizures [19]. Discovery of novel regulatory molecules for PHGDH may provide potential cures for these diseases.

Allostery is an essential biological regulatory mechanism, and allosteric regulation can be achieved by various allosteric effectors, ranging from small molecules to macromolecules. Merdanovic *et al.* [20] summarized known allosteric effectors for proteases and their possible allosteric mechanisms. Traditional chemical biology and drug design efforts are focused on the discovery of small molecules directly targeting substrate-binding sites, and the recognition of allosteric sites as potential targeting sites presents an entirely new way to design diverse inhibitors, or even activators. Methods for allosteric site identification and regulator design have been developed. For example, Wells *et al.* developed the tethering method for allosteric molecule discovery, and discovered novel allosteric inhibitors for caspase-3 and -7, and allosteric activators for procaspase-3 and -7 [21], [22]. Compared to experimental methods, the numbers of computational methods developed for allosteric site detection and regulator design are limited.

**Figure 1:**
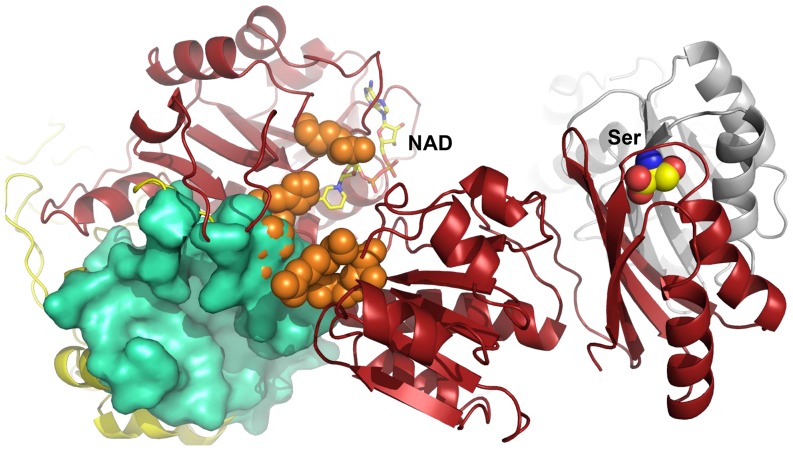
Structure of site I in PGDH (PDB code: 1YBA). Site I is represented by the green surface, the active site is indicated by orange spheres, and the cofactor NAD^+^ and the endogenous allosteric L-serine are illustrated in stick and sphere, respectively.

In a previous study, we developed a method for allosteric site prediction based on a two-state Gō model and used it to predict potential novel allosteric sites in *E. coli* PGDH [23]. Two potential allosteric sites were identified, one is close to the active site and the nucleotide binding site (Site I) (Figure 1) and the other is near the regulatory domain (Site II). Both sites are larger than the L-serine allosteric site and may accommodate more diversified allosteric effectors. Three novel inhibitors have been identified targeting Site II [23]. Activators will provide a new dimension, in addition to inhibitors, for the regulation of the L-serine synthetic pathway. In the present study, using site I as a potential allosteric site, we discovered novel allosteric activators as well as inhibitors using virtual screen, enzymatic bioassays, surface plasmon resonance (SPR) assay and mutagenesis studies.

## Materials and Methods

### Materials

Reagents for molecular cloning, protein expression and purification, enzyme assay and mutagenesis experiments were prepared as previously described [23]; sensor surfaces and other consumables for Surface Plasmon resonance (SPR)-based assays were purchased from GE Healthcare Biacore (GE Healthcare Biacore, Uppsala, Sweden); compounds **1-3** and their analogs were purchased from SPECS [24]. The purity of compounds **1-3** and their analogs from SPECS database is more than 90% and for most compounds greater than 95% (reconfirmed by LC-MS, and the date were in accordance with that available through the SPECS web site).

### Allosteric Site Prediction

Allosteric site prediction was performed as reported in a previous publication [23]. Briefly, an initial ensemble that favored the unbound state of PGDH was constructed using the two-state Gō model. Then, perturbations were added to the target site to simulate the binding of a ligand. If the perturbations caused a population shift of the ensemble, i.e., the new ensemble favored the bound state, the target site was predicted to be an allosteric site. Two possible allosteric sites, I and II, were found for PGDH. Compared to the known Ser allosteric site, both site I and site II have larger volumes and are more suitable for designing novel effectors. Site II is located near the regulatory domain and inhibitors targeting this site have been identified. Site I is close to the active site and the nucleotide binding site, with a volume of 1628.0 Å^3^ and a predicted maximal pK_d_ of 9.96. It shared the residue Lys141 with the active site.

### Molecular Docking

Molecular docking studies were carried out using a similar procedure for identifying allosteric effectors for site II [23]. Firstly, rigid body docking was performed with default parameters using the program DOCK 6 [25] to screen the SPECS library. Secondly, flexible ligands and rigid receptor docking was performed using the program Autodock Vina [26] with default parameters to further screen the top 10000 compounds from the DOCK results. Thirdly, the binding conformations of the top 1000 from Autodock Vina were exported and manually selected according to the following criteria: (1) The compound in the pocket had at least 90% occupancy ratio. (2) The compound formed at least two hydrogen bonds. (3) The compound was not a polypeptide (4) The compound did not contain metal atoms. And finally 170 compounds were manually selected and purchased from SPECS.

### Molecular Cloning, Protein Expression and Purification

Plasmid construction, protein expression, and purification were performed as previously described [23]. The PGDH coding region was ligated into the pET21a(+) vector and transformed to the BL21 (DE3) strain of *E. coli*. Recombinant cells were cultivated at 37°C until the OD_600_ reached 0.6-0.8. Then, PGDH expression was induced and the cells were grown for another 4 h at 30°C. Cells were harvested by centrifugation (6000 rpm, 15 min) and broken by sonication. Insoluble material was separated by centrifugation (17000 rpm, 30 min) and the supernatant was purified using a nickel-nitrilotriacetic column and then a gel-filtration column.

### Enzyme Assay

Enzyme activity was measured by monitoring the NADH to NAD^+^ change in fluorescence emission at 456 nm (excitation at 338 nm) in the presence of enzyme and α-ketoglutarate [23]. α-Ketoglutarate is a substrate analog for *E. coli* PGDH and was used in these experiments since it is more easily obtained and more stable than the physiological substrate, hydroxypyruvic acid phosphate [27].

To evaluate the effects of compounds on PGDH activity, compounds were first examined to have no fluorescence absorption at 456 nm, and then pre-incubated with enzyme samples in the assay buffer (20 mM tris-(hydroxymethyl) aminomethane hydrochloride (Tris-HCl), pH 7.5, 1 mM DTT, 1 mM EDTA, and 0.25 mM NADH) for 6 min at 25°C. Each compound was dissolved in DMSO at a final concentration of 5%, which did not affect the assay signal. Fluorescence signals were recorded for 3 min with a kinetics mode program. The cofactor concentration in the assay buffer was 0.25 mM and the substrate concentration was 5 mM [28]. IC_50_ or AC_50_ values were obtained by fitting the data to a three-parameter Hill model of the graph of log dose against percentage inhibition or activation from at least three sets of experiments. Percentages of inhibition or activation were calculated according to the following equation: 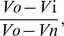
in which V_o_ and V_i_ represent the maximum reaction rate of the enzyme incubated without or with compounds and V_n_ represents the maximum degradation rate of NADH.

### Competition Experiments

To investigate competition effects between the compounds and the substrate α-ketoglutarate, we performed compound-substrate competition experiments as follows:

Before α-ketoglutarate was added to start the reaction, the enzyme sample was pre-incubated with the cofactor and the compound for 6 min at 25°C. The compound was kept at a constant inhibitory concentration, while the substrate concentration was gradually increased from 78 µM to 625 µM. To further study relationships between the inhibition rate of compounds and the substrate concentration, for compounds **1** and **2**, the substrate concentration was increased to 10 mM. To determine the effect of compounds on PGDH activity at lower substrate concentrations, 50, 50, and 130 µM of compounds **1**, **2**, and **3**, respectively, were used. At these concentrations, the compounds inhibited PGDH activity by ∼50% when the substrate concentration was 5 mM. The cofactor NADH concentration was kept constant at 0.25 mM.

### Kinetic Analysis

As in the competition experiments, enzyme reaction velocity vs. substrate relationships were measured at various compound concentrations. The compound concentrations used were 120, 80, 40, 24, 8, 4.8, 1.6, and 0.32 µM.

### SPR Experiments

The binding affinity of compounds **1** and **2** towards PGDH and its mutants were assayed using the SPR-based Biacore T200 instrument. PGDH and its mutants were immobilized on a CM5 sensor chip by using standard amine-coupling at 25°C with running buffer HBS-P (20 mM phosphate buffer, 2.7 mM NaCl, 137 mM KCl, 0.05% surfactant P-20, pH 7.4), respectively. The carboxyl groups of the sensor surface were activated by injection of a solution containing 0.2 M N-ethyl-N′-(3-dimethylaminopropyl) carbodiimide (EDC) and 0.05 M N-hydroxysuccinimide (NHS), PGDH in 10 mM sodium acetate buffer (pH 4.5) was then injected at a flow rate of 10 µl/min to couple to the sensor surface, and the remaining active sites in the flow cell were finally blocked by 1M ethanolamine (EA). A reference flow cell was activated and blocked in the absence of PGDH. In the direct binding experiments between PGDH and compounds **1** and **2**, PGDH immobilization level was fixed at 800 response units (RU), and then different concentrations of compounds **1** and **2** containing 5% DMSO were serially injected into the channel to evaluate binding affinity. Regeneration was achieved by extended washing with the running buffer after each sample injection. The equilibrium dissociation constants (K_D_) of the compounds **1** and **2** were obtained by fitting the data sets to 1∶1 Langmuir binding model using Biacore T200 Evaluation Software. In the binding site verification SPR experiments, the sensor chip surface was immobilized with WT PGDH and its mutants to a RU of 4000, respectively. The analytes, compounds **1** and **2**, were injected over the chip at a fixed concentration of 50 and 25 µM, respectively.

### Mutagenesis Experiments

All mutagenesis experiments were carried out according to the instructions of the QuikChange site-directed mutagenesis (Stratagene). The plasmid pET-21a(+)-containing wild-type (WT) PGDH was mutated to obtain the mutants E129A, S146A, F147A, K256A, K152AK230A, and E129AK256A. The DNA sequences of all mutants were verified by DNA sequencing. The protein expression and activity assays of the mutants were performed as described for the WT.

## Results

### Identification of Novel Regulatory Molecules

**Figure 2:**
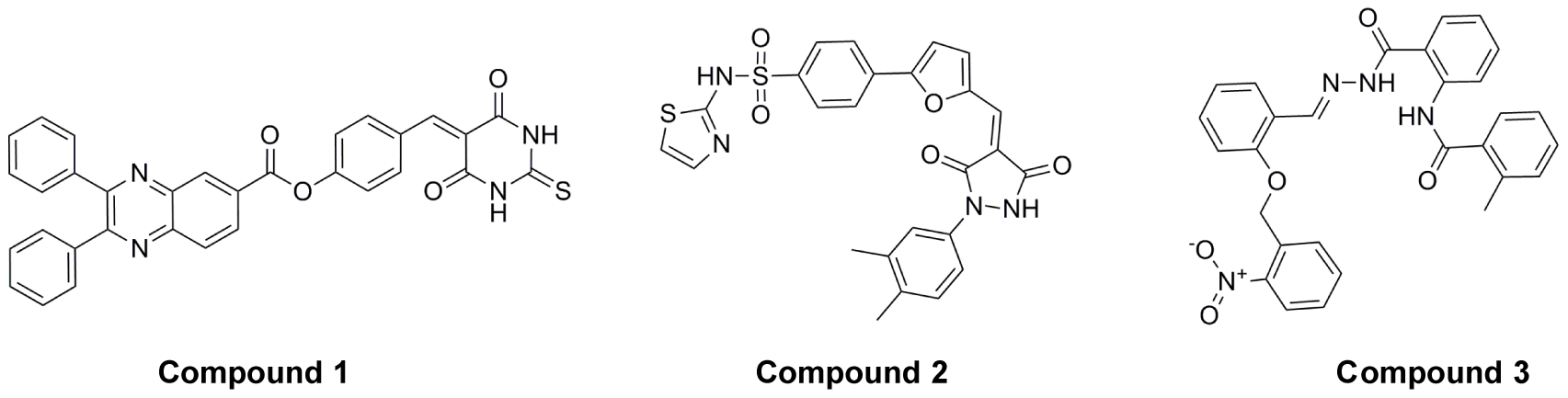
Structures of the compounds 1-3. The SPECS IDs of compounds **1**-**3** are AN-698/40677526, AN-023/41981714 and AG-205/07681005.

**Figure 3:**
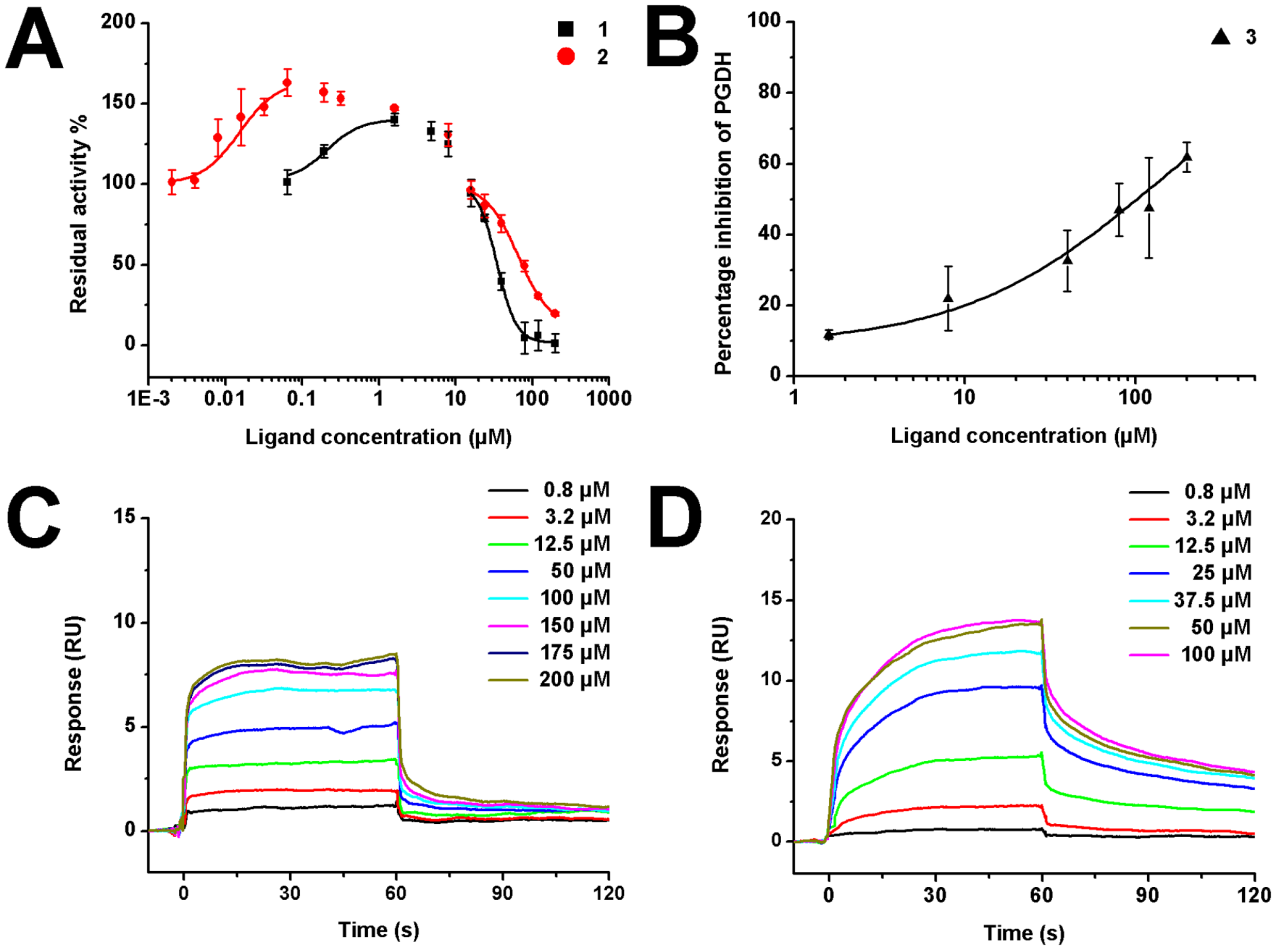
Dose-response curves of compounds 1-3. (A) Residual enzyme activity versus compound concentration. The IC_50_ values were 34.8±1.3 for compound **1**, and 58.0±9.0 µM for compound **2**. The AC_50_ value was 34.7±4.5 nM for compound **2.** (B) Enzyme inhibition dose-response curves of compound **3.** The fitted IC_50_ value was 131±12 µM. (C–D) SPR dose-response curves of compound **1**, compound **2** with immobilized PGDH, respectively. The K_D_ values were 42.6±2.1 for compound **1** (C), and 19.0±1.9 µM for compound **2** (D). K_D_ values of the compounds **1** and **2** were obtained by fitting the data sets to 1:1 Langmuir binding model using Biacore T200 Evaluation Software. Residuals for all SPR sensorgrams were less than 2 RU. Chi-square values were 2.4 for C and 1.2 for D.

After virtual screen, we purchased 170 compounds and tested their ability to regulate PGDH activity. Fifteen compounds showed inhibition; three of them significantly affected PGDH activity. The chemical structures of these three compounds are shown in Figure 2. The IC_50_ values for compounds **1** to **3** were 34.8±1.3, 58.0±9.0, and 131±12 µM, respectively (Figures 3A and 3B). Due to solubility restriction, the IC_50_ value for compound **3** was obtained from a partial dose-inhibition curve (maximal inhibition rate 61.9%), which may only be partially reliable.

Interestingly, compounds **1** and **2** also showed a partial activation phenomenon at low concentration (lower than 16 µM). We plotted the residual activity versus compound concentration (log) at the substrate concentration of 5 mM (Figure 3A). Residual activity larger than 100% indicates activation, while lower than 100% means inhibition. At concentrations larger than 16 µM, both compounds were inhibitory. However, at the lower concentration range (less than 16 µM), both of them can increase the enzyme activity. Compound **2** increased the enzyme activity to a maximum of 163%, while the activating ability for compound **1** was about 140%. Compound **2** started to activate the enzyme at a concentration of about 2 nM and reached the highest activation at about 64 nM. The enzyme activity then began to go down resulting from the mixed effects of activation and inhibition in the concentration range of 64 nM to 1.6 µM. When the concentration increased above 1.6 µM, compound **2** behaved like ordinary inhibitors. Compound **1** showed a similar behavior: 64 nM-1.6 µM activation, 1.6 µM-16 µM mixed effects, >16 µM inhibition. By fitting the activation dose response curve, the AC_50_ value for compound **2** was 34.7±4.5 nM.

To further eliminate false positives and validate that compounds **1** to **3** were true hits, SPR technology was used to determine their direct binding affinity. After PGDH was immobilized on the CM5 chip, serial concentrations of compounds **1** to **3** were injected automatically. The binding signals were continuously recorded in response units (RU) and presented graphically as a function of time. A reliable K_D_ value for compound **3** was not got due to its weak binding ability. As shown in Figures 3C and 3D, compounds **1** and **2** bound to PGDH in a concentration-dependent manner, K_D_ values were 42.6±2.1 for compound **1** and 19.0±1.9 µM for compound **2**. SPR experiments also demonstrated that these compounds did not aggregate at the experimental condition.

### Binding Site Verification

To test whether these compounds indeed bound to site I and acted as allosteric effectors, we performed compound-substrate or -serine competition experiments, mutagenesis studies and SPR assays.

**Figure 4:**
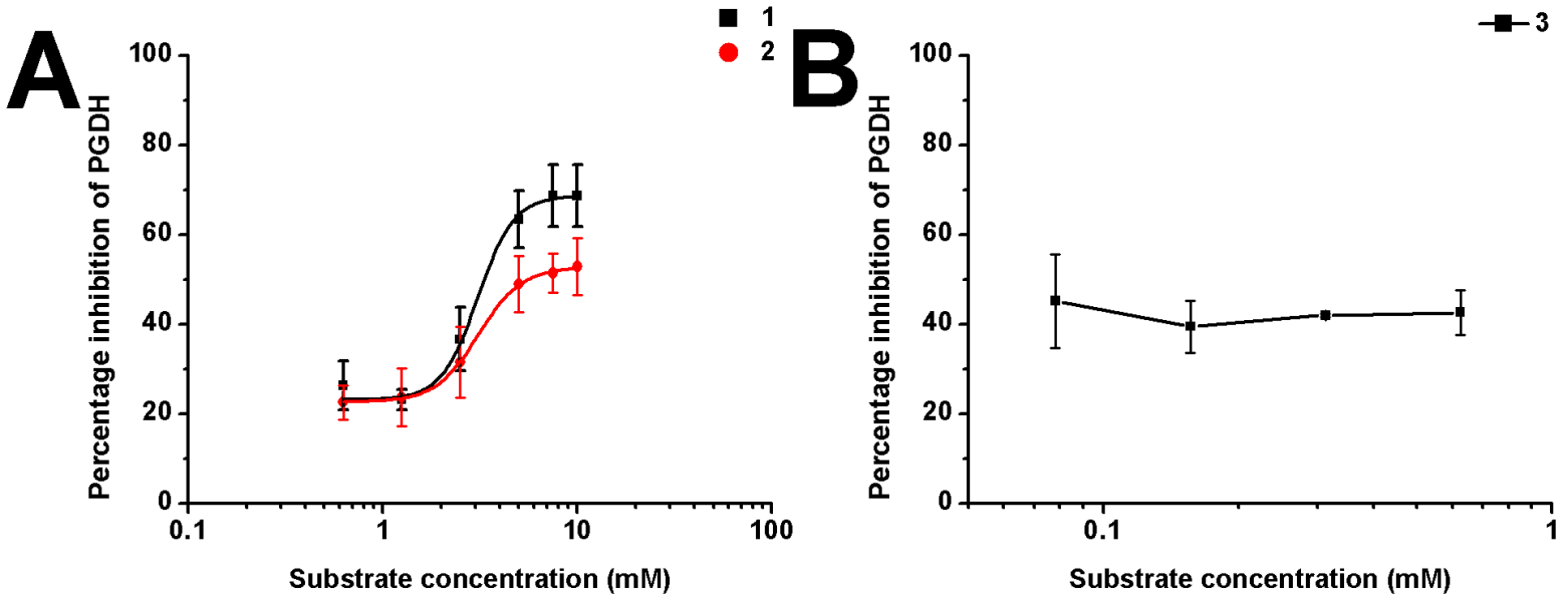
Competitive assay of compounds 1-3 with the substrate. (A) Compounds **1** and **2** and the substrate do not competitively bind to the same site. Increasing the substrate concentration led to higher inhibition rates of the compounds in contrast to lowered inhibition as expected for competitive inhibitors, indicating that these compounds do not bind to the substrate-binding site. (B) Substrate competition curve of compound **3**. The percentage inhibition did not change along with the increase of substrate concentration, indicating that there are no significant interactions between compound **3** and the substrate binding site.

Compound-substrate competition can be explained as follows: if the compound and the substrate competitively bind to the same site and the compound concentration is kept constant, increasing the substrate concentration will impair the binding ability of the compound, thereby decreasing its inhibitory effect. No substrate competition was observed for compounds **1-3** (Figures 4A and 4B). For compounds **1** and **2**, along with the increase of substrate concentration from 156 µM to 10 mM, the percentage of inhibition increased from 20% to 50% or much higher. This demonstrates that a cooperative effect exists between compound binding and substrate binding.

Likewise, serine-compound competition experiments indicated that the ability of serine to decrease the maximal reaction rate of the enzyme was not significantly influenced by compounds **1** and **2**, when the concentrations were kept at 40 µM (Figure S1 in File S1).

**Figure 5:**
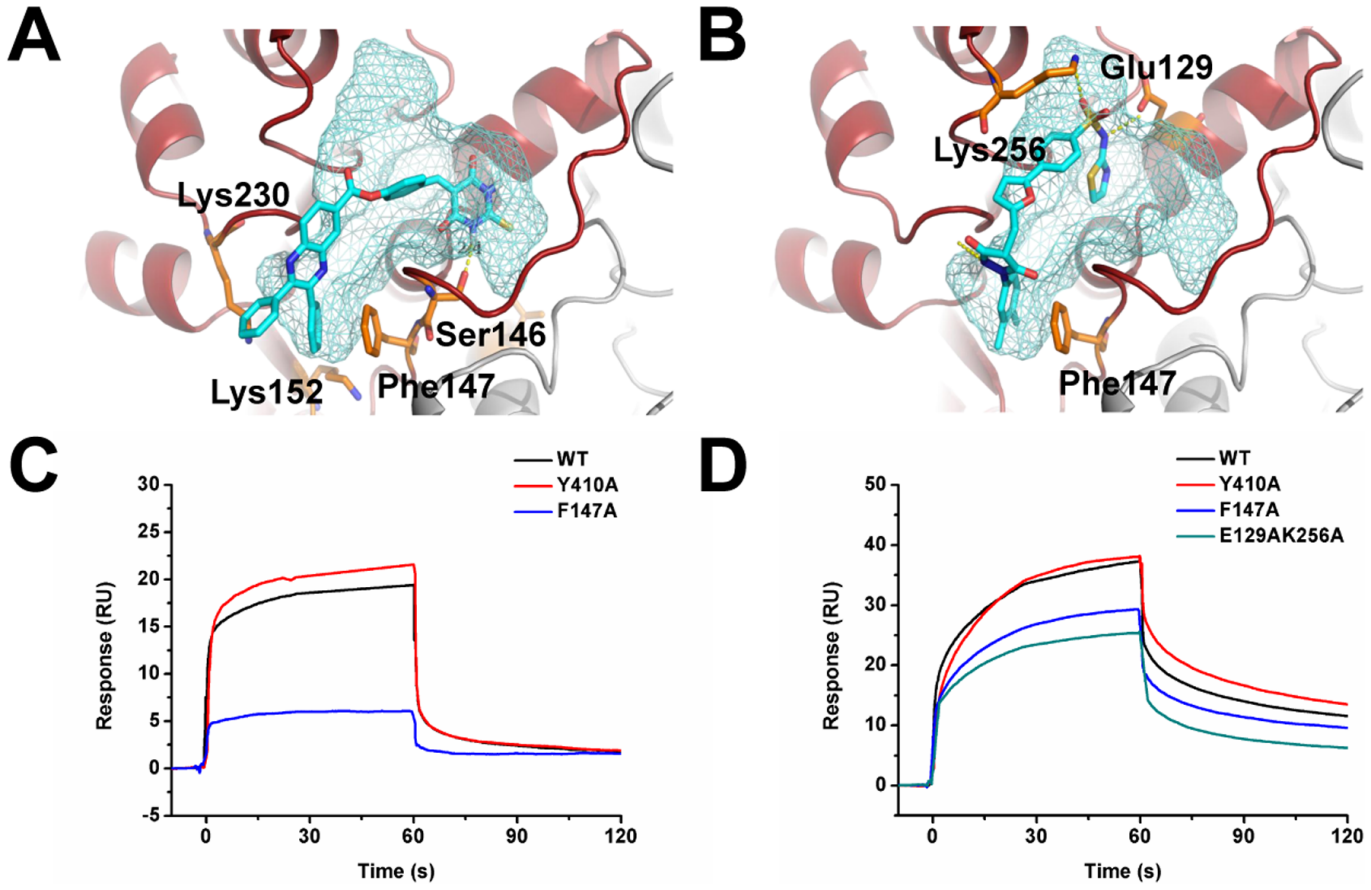
Binding site verification. (A–B) The complex structure model of compounds **1** and **2** binding to site I in PGDH. Compound **1** (A), compound **2** (B), and the mutation sites are shown in stick representation, while site I is in surface representation. (C–D) SPR direct binding curves of WT, Y410A, F147A and E129AK256A. WT PGDH and its mutants were immobilized on the sensor chip. Compounds **1** (C) and **2** (D) were injected over the chip at a fixed concentration of 50 and 25 µM, respectively.

To further confirm that the compounds bind at the predicted site, we carried out mutagenesis studies at site I and measured the effect of the mutants on the two strong compounds, **1** and **2**. Based on the docking structures, mutants S146A, F147A, and K152AK230A for compound **1** and mutants E129A, F147A, K256A, and E129AK256A for compound **2** were tested (Figures 5A and 5B). All the mutants retained their enzymatic activity (Figure S2 in File S1), and the interactions between the compounds and the mutants were much weaker than those with the WT (Figure S3 in File S1). For example, compound **2** exhibited IC_50_ values >200 µM (Table 1) with mutants K256A and E129AK256A.

**Table 1:** Mutation effects on the inhibition rates of compounds.

		IC_50_ values for mutants (µM)					
compound	WT^a^	E129A	S146A	F147A	K256A	K152A K230A	E129A K256A
1	34.8±1.3	ND^b^	175±88	181±26	ND	>200	ND
2	58.0±9.0	99±13	ND	158±22	>200	ND	>200

a IC_50_ values for wild type (µM).

b ND, not determined, based on the docking results.

We also tested whether compounds **1** and **2** might interact with the other allosteric site (site II) we discovered before [23]. The key binding residue in site II, Y410 was mutated to A. The binding of these two compounds with the wild type enzyme, Y410A, and two site I mutants (F147A, E129AK256A) were tested using SPR direct binding assay. The immobilization levels for WT, Y410A, F147A and E129AK256A were kept almost the same (3506, 3792, 3756 and 3600 RU, respectively) and the concentrations of compounds **1** and **2** were kept at 50 µM and 25 µM. As shown in Figures 5C and 5D, the site II mutant, Y410A, retained the same extent of compound binding ability as WT, while the site I mutants, F147A and E129AK256A, showed a large decrease in compound binding ability. This verified that both compounds **1** and **2** bind specifically to the site I as designed.

### Influence of Allosteric Effector Binding on the Enzyme Kinetics

**Figure 6:**
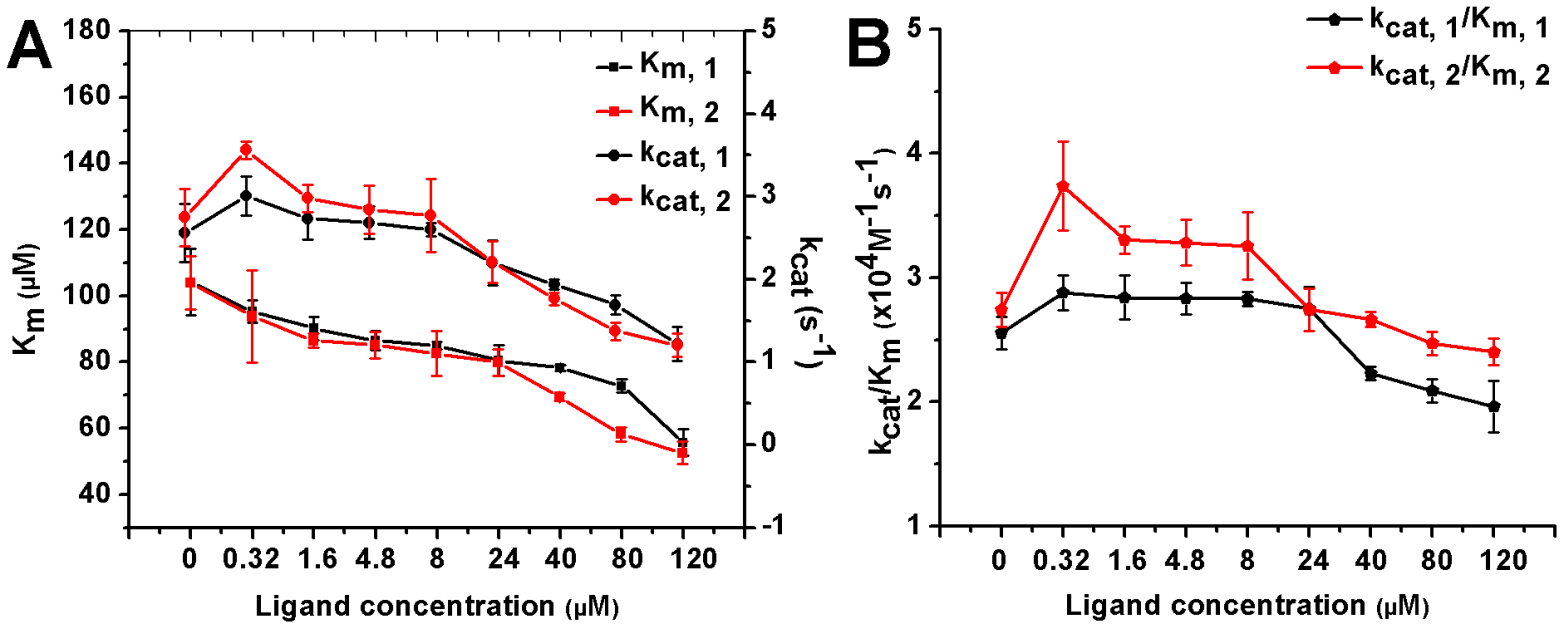
The K_m_, k_cat_ and k_cat_/K_m_ values versus compound concentration. The kinetic data for PGDH show that the values of K_m_ decrease with increasing concentrations of **1** and **2** ranging from 0 µM to 120 µΜ, while values of k_cat_ and k_cat_/K_m_ show an initial increase and then a decrease.

To evaluate the effects of the compounds on enzyme kinetics, Michaelis-Menten plots of PGDH incubated with different concentrations of compounds were measured. The apparent kinetic parameters k_cat_ and K_m_ were estimated by non-linear curve-fitting using Graphpad Prism 5.0 software, and their values are shown in Figure 6. With increasing concentrations of added compounds from 0 µM to 120 µM, the K_m_ of the enzyme decreased steadily, whereas the k_cat_ and the k_cat_/K_m_ of the enzyme showed an initial increase, followed by a decrease. In our activity assay, the K_m_ value of pure enzyme was about 100 µM, larger than any K_m_ values obtained from the enzyme incubated with compounds. In other words, compounds **1** and **2** enhanced the substrate affinity for the enzyme. The k_cat_ of the pure enzyme was about 2.6 s^−1^, which was in between the k_cat_ values of the enzyme at the molecular concentrations of 8 µM and 24 µM.

### Structure-Activity Relationship (SAR) Study for Compound 1

Analogs of compound **1** were identified, purchased, and tested. The similarity profile of compound **1** was computed for SPECS using PHASE, a highly flexible system that can be used for 3D database searching [29]. Individual compounds with high similarity to the reference compound were subjected to docking studies [30]. Twenty-five compounds with good docking scores were selected for experimental testing.

Among the 25 compounds, 5 showed inhibition activities (The chemical structures and the dose-response curves of these five compounds are shown in Figures S4 and S5 in File S1), though with lower activities compared to the parent compound. We further verified their binding sites using compound-substrate competition experiments (Figure S6 in File S1).

Molecular modeling studies indicated that the high potency was largely due to two key interactions within the pocket: (i) π-π stacking between the phenyl group of the compounds and the side chain of F147, and (ii) H-bonds between the polar groups of compounds and polar residues in site I (Figure S7 in File S1). Poor π-π stacking or a smaller number of H-bonds will deteriorate the interactions.

### SAR Study for Compound 2

The same approach was used for the SAR study of compound **2**. Twenty-five compounds with a good similarity profile and high docking scores were selected and tested; two of them showed inhibition activities (The chemical structures and the dose-response curves of these two compounds are shown in Figures S4 and S8 in File S1). The IC_50_ values of compounds **2-1** and **2-2** were 96±22 and 22.3±2.5 µM, respectively. The inhibition activity for compound **2-2** was higher than that of compound **2**. Compound-substrate competition experiments showed that both compounds, **2-1** and **2-2,** are substrate independent.

Key interactions between analogs of compound **2** and site I include π-π stacking with the side chain of F147 and H-bonds with the polar residues (Figure S9 in File S1). Compared with the docking result of compound **2**, compound **2-1** had weak π-π stacking with F147 and equal numbers of H-bonds with polar residues, resulting in decreased activity. For compound **2-2**, though it lacked a H-bond with K256, it had a smaller distance of 4.12 Å to F147 and an additional cation-π interaction with K256. The side chain amino group of K256 was located just above the benzene ring of compound **2-2**, and the distance between the two was 4.55 Å. Early in 1986, Burley and Petsko examined 33 high resolution (2 Å or higher), refined protein crystal structures and demonstrated that positively charged or δ (+) amino groups, like lysine and arginine, tend to be located within 6 Å of the ring centroids of aromatic amino acids, where they form van der Waals' contacts with the δ (-) π-electrons [31]. Although solvent water molecules can affect the cation-π interaction, its strength was still considerable with the H-bonds in aqueous solution [32]. Considering the combined effect of the compounds and the residues in the pocket, compound **2-2** showed better activity than compound **2**, as manifested in the IC_50_ values.

## Discussion

We have discovered three novel allosteric effectors and seven of their analogs for PGDH based on the predicted allosteric site I. These compounds were confirmed to bind to the predicted site by compound-substrate competition and mutagenesis studies, demonstrating that site I is indeed a novel allosteric site. Though the activities of compounds **1** and **2** were comparable to the endogenous allosteric effector, L-serine (which has an IC_50_ of 8 µM) [33], the large size of site I is more suitable for further compound optimization and the discovery of novel allosteric effectors with diverse chemical structures.

Our enzyme kinetic analysis showed that increasing the concentration of the compound led to a dose-dependent decrease of the apparent Michaelis constant (K_m_) of the enzyme, while the k_cat_ and k_cat_/K_m_ values exhibited an initial increasing and then a decreasing trend. A previous study has indicated that the enzyme can be described as a dimer of dimers [3], and it is likely that only two active sites across the diagonal of the tetramer are functional at a given time [6]. We proposed a possible mechanism to explain the concentration dependent influences of the compounds on the enzyme kinetic parameters. In terms of a dimer, when the concentration of the compound is low, the compound may only bind to one pocket, enhancing substrate binding at the adjacent active site and weakening the efficiency of the catalytic process at this active site. Meanwhile, the compound can induce a conformational change in the enzyme, and this would be favorable for substrate binding to the active site of the other subunit and would also increase catalytic efficiency. Thus, the compound becomes an activator, as exemplified by the k_cat_/K_m_ values obtained at low molecule concentrations. When the concentration of the compound is sufficiently high, it will occupy site I in both subunits and decrease the k_cat_ and k_cat_/K_m_ values for both active sites, thus becoming an inhibitor. We have built an explanatory model for the dimer to show that this scenario was compatible with our experimental findings to a certain extent. Following the kinetic mechanisms in Scheme S1 in File S1 and the manually set parameters, the simulation result shown in Figure S10 in File S1 shows the correct dose-response curve with the properties of low-concentration activation and high-concentration inhibition. An attempt to fit the model to the experimental data was not very successful, possibly due to the unaccounted dimer-dimer interactions.

From 1960s to date, PGDH enzymes were studied exhaustively in *E. coli*, particularly for their catalytic and serine allosteric inhibition mechanisms. Early studies [2] indicated that a variety of amino acids and analogs of L-serine, such as glycine and threonine, could also inhibit the enzyme, but led to an increase in the inhibition concentration from micromolar to millimolar. More precisely, only L-serine exhibits a micromolar binding constant. In the present study, 10 novel allosteric inhibitors with micromolar activity were discovered, which all bound to the predicted allosteric site I.

Slaughter *et al.* [34] reported in 1975 that PGDH, the first enzyme of serine biosynthesis in peas [35], [36], is specifically and markedly activated by L-methionine. L-methionine reaches maximal activation at 10 mM and causes a four- to five-fold increase in enzyme activity. However, the amino acid failed to stimulate PGDH enzymes in other organisms, indicating it is not a general activator. To date, L-methionine is still the only activator reported for the family of PGDH enzymes with unknown binding sites. We have discovered that compounds **1** and **2** can activate *E. coli* PGDH at low concentrations and become inhibitors at high concentrations. The AC_50_ value of compound **2** is 34.7 nM, which is much lower than that of L-methionine for PGDH from pea.

Overall, including the serine-binding site and the two sites discovered in our previous [23] and current study, three completely different allosteric sites in PGDH were discovered. Three different allosteric sites have rarely been reported in a single enzyme before. One example was reported by Ulaganathan *et al.* [37], in which the inhibitor BI8 was found to bind not only to the well-characterized ispinesib-binding site, but also to a novel allosteric site in human kinesin Eg5. Targeting multiple allosteric sites simultaneously using either combinations of ligands or multiple-binding site ligands may present another promising strategy for strong potency and selectivity.

To conclude, we have identified a novel allosteric site in *E.coli* PGDH and discovered both activators and inhibitors targeting this site. Since a regulatory superstructure exists with various metabolic pathways [38], the newly discovered PGDH allosteric effectors may can be used for the regulation of the *E.coli* amino acid synthesis pathway. The allosteric sites discovered by this method also open up new possibilities for metabolic network regulation.

## Supporting Information

File S1 **Supplementary Material and Methods and Figures.**
(PDF)
