# Supplementary material for: Discovery of Novel Allosteric Effectors Based on the Predicted Allosteric Sites for Escherichia coli D-3-Phosphoglycerate Dehydrogenase
Source: PLoS One. 2014 Apr 14;9(4):e94829. doi: 10.1371/journal.pone.0094829 (PMC3986399; doi:10.1371/journal.pone.0094829)
Supplement: File S1 — Supplementary Material and Methods and Figures. (PDF) [file pone.0094829.s001.pdf]

Discovery of Novel Allosteric Effectors Based on the Predicted Allosteric Sites for *Escherichia Coli* D-3-Phosphoglycerate Dehydrogenase

Qian Wang, Yifei Qi, Ning Yin, Luhua Lai

Supporting Information

Material and Methods

Serine-compound competition experiment

Serine-compound competition experiments were did like that for compound-substrate experiments, before the reaction was started, the enzyme was pre-incubated with the mixture of serine and compound for 6 minutes. During this process, compound was kept at a constant inhibitory concentration, and the serine concentrations were changed from 1.6 to 200 μM. The concentrations of compounds **1** and **2** were selected as 40 μM.

Competition experiments for analogs and the substrate

For analogs of compounds **1**, the concentrations of compounds **1-1**, **1-2**, and **1-3** were kept at 92, 100, and 120 μM, while gradually increasing the substrate concentration from 78 μM to 625 μM. Since compounds **1-4** and **1-5** began to precipitate when their concentrations were above 100 μM, competition experiments for them were not performed. Compounds **1-2** and **1-3** showed substrate independent inhibition activity, while **1-1** can be influenced by the substrate, with the inhibition rate changing from -16% to 48%. For analogs of compound **2**, the concentrations of compounds **2-1** and **2-2** were 75 and 15 μM, and the experimental results indicated that compounds **2-1** and **2-2** did not interacted with the active site.

The binding mode of compounds

All the compounds were re-docked by Glide based on the two stages of the docking protocol, Standard Precision (SP) and Extra Precision (XP). First stage of SP docking was used to find probable good binding mode, and the SP resultant compounds were then docked using more accurate XP mode.

Computational simulation for compounds 1 and 2

A simplified model was built to explain the low-concentration activating and high-concentration inhibiting phenomena exemplified by compounds **1** and **2** toward PGDH. Since PGDH can be largely considered as a dimer of dimers, we have built our model for only one dimer upon the assumption of the independence of the two dimers. As explained in main text, allosteric binders could enhance the affinity of PGDH for its substrate, but this kind of cooperative binding is disadvantageous to the catalytic process. We ran an example simulation following Scheme 1 with manually set parameters, and the result was qualitatively consistent with the experimental observations. An attempt to fit the model to the experimental data was not very successful, possibly due to the interaction between the dimers and other subtleties which could not be fully addressed in such a simple model.

Supporting Figures

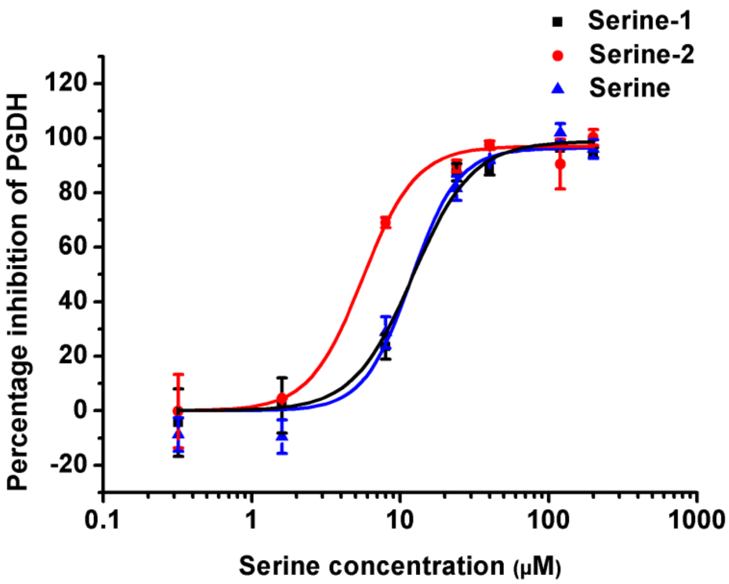

Figure S1. Compounds **1** and **2** had no significant effect on L-serine inhibitory ability. The IC_{50} values were 11.8 ± 0.9 μM for serine with the presence of compound **1**, 5.6 ± 0.3 μM for serine with the presence of compound **2**, and 12.2 ± 1.3 μM for serine alone.

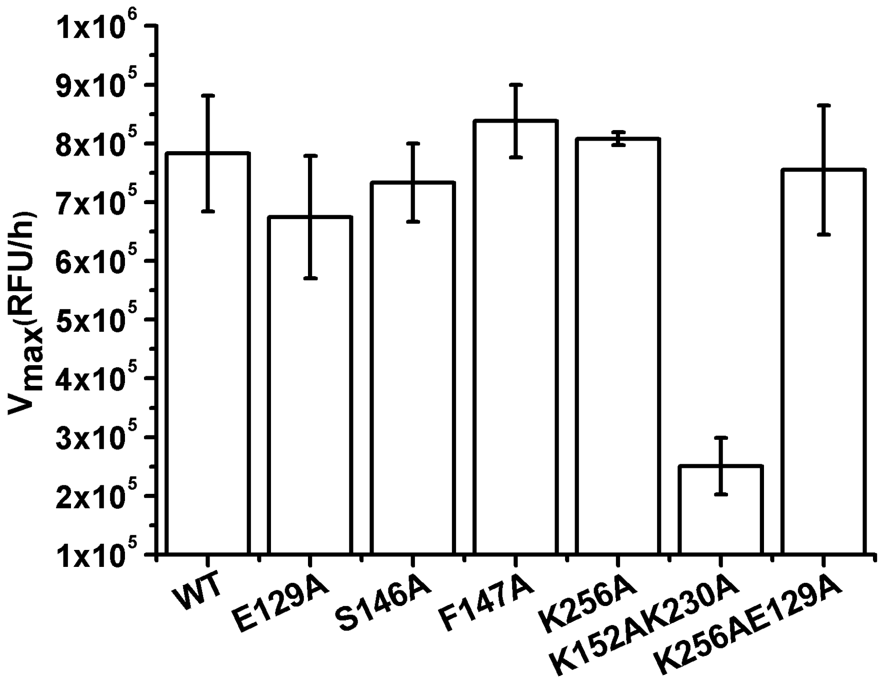

Figure S2. None of the mutations at site I caused PGDH to entirely lose its activity. The kinetic method was used to measure the maximum reaction rate (V_{max}) by monitoring the fluorescence emission of NADH at 456 nm (RFU, relative fluorescence units).

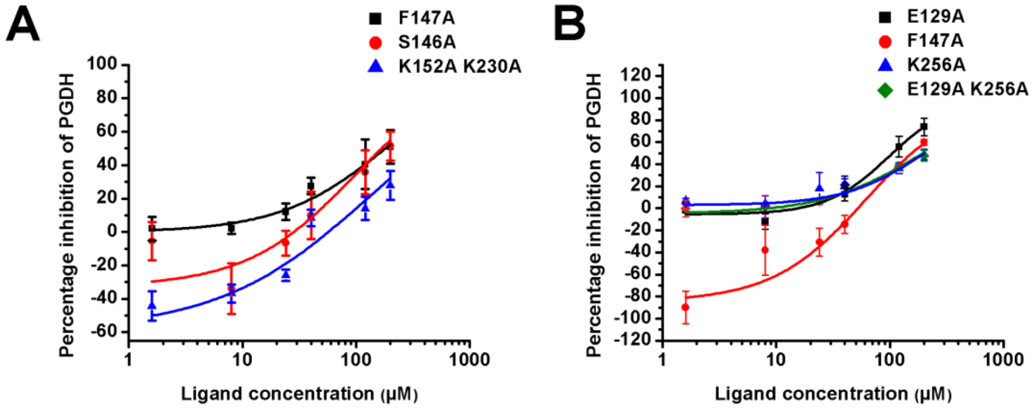

Figure S3. Dose-response curves for compounds 1 and 2 with mutations in site I. Three mutants (F147A, S146A, K152AK230A) for compound **1** (A) and four mutants (E129A, F147A, K256A, E129AK256A) for compound **2** (B) weakened their inhibition rate, suggesting that they bind at site I, in accord with the docking results (Figures 5A and B).

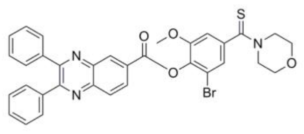

Compound 1-1

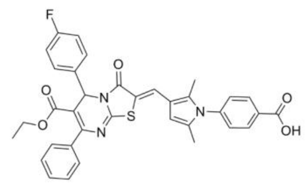

Compound 1-2

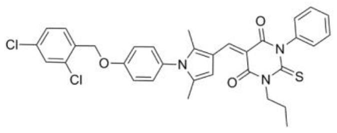

Compound 1-3

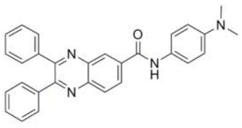

Compound 1-4

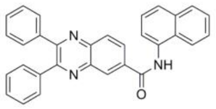

Compound 1-5

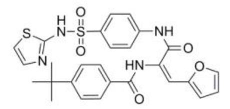

Compound 2-1

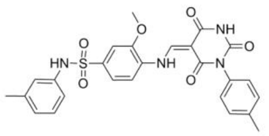

Compound 2-2

Figure S4. Structures of the analogs. The SPECS IDs of compounds **1-1**, **1-2**, **1-3**, **1-4**, **1-5**, **2-1** and **2-2** are AN-698/40861731, AN-648/15596193, AH-487/41184356, AG-690/10410040, AN-698/40861706, AG-690/12243007 and AK-968/12117152.

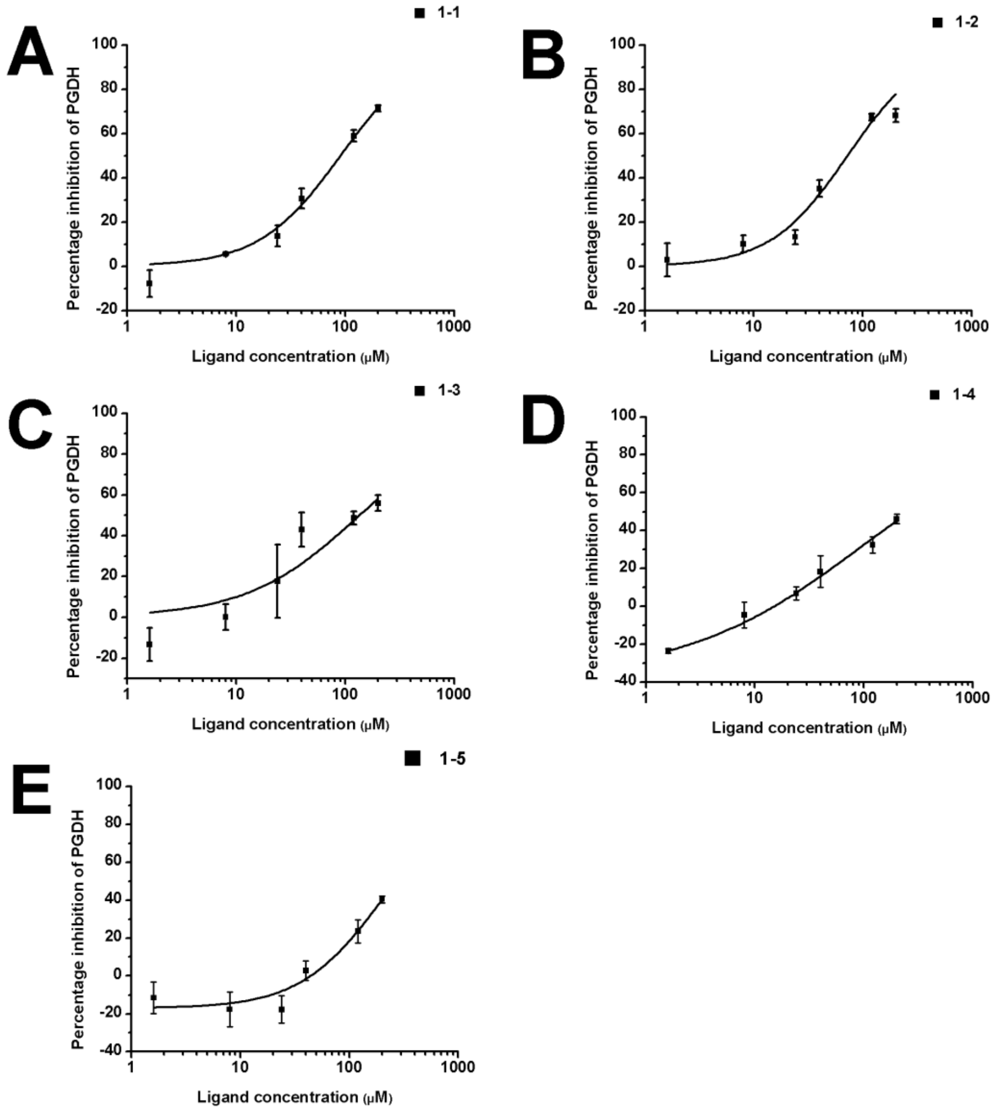

Figure S5. Dose-response curves for analogs of compound 1. Inhibition experiments showing that the IC_{50} values of compounds **1-1** to **1-5** were 72 ± 22 for **1-1**, 90.2 ± 3.5 for **1-2**, 135 ± 23 for **1-3**, 215 ± 15 for **1-4**, and 243 ± 34 μM for **1-5**.

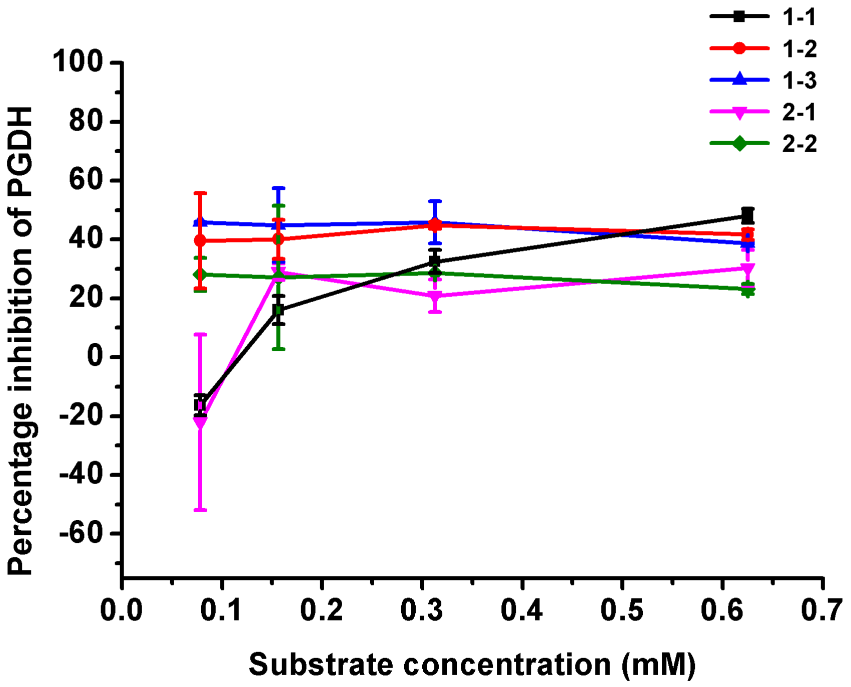

Figure S6. Competition experiments for analogs and the substrate. Substrate competition curves showing that compounds **1-1** interacted with the substrate binding site, while **1-2**, **1-3**, **2-1**, and **2-2** did not show obvious interactions.

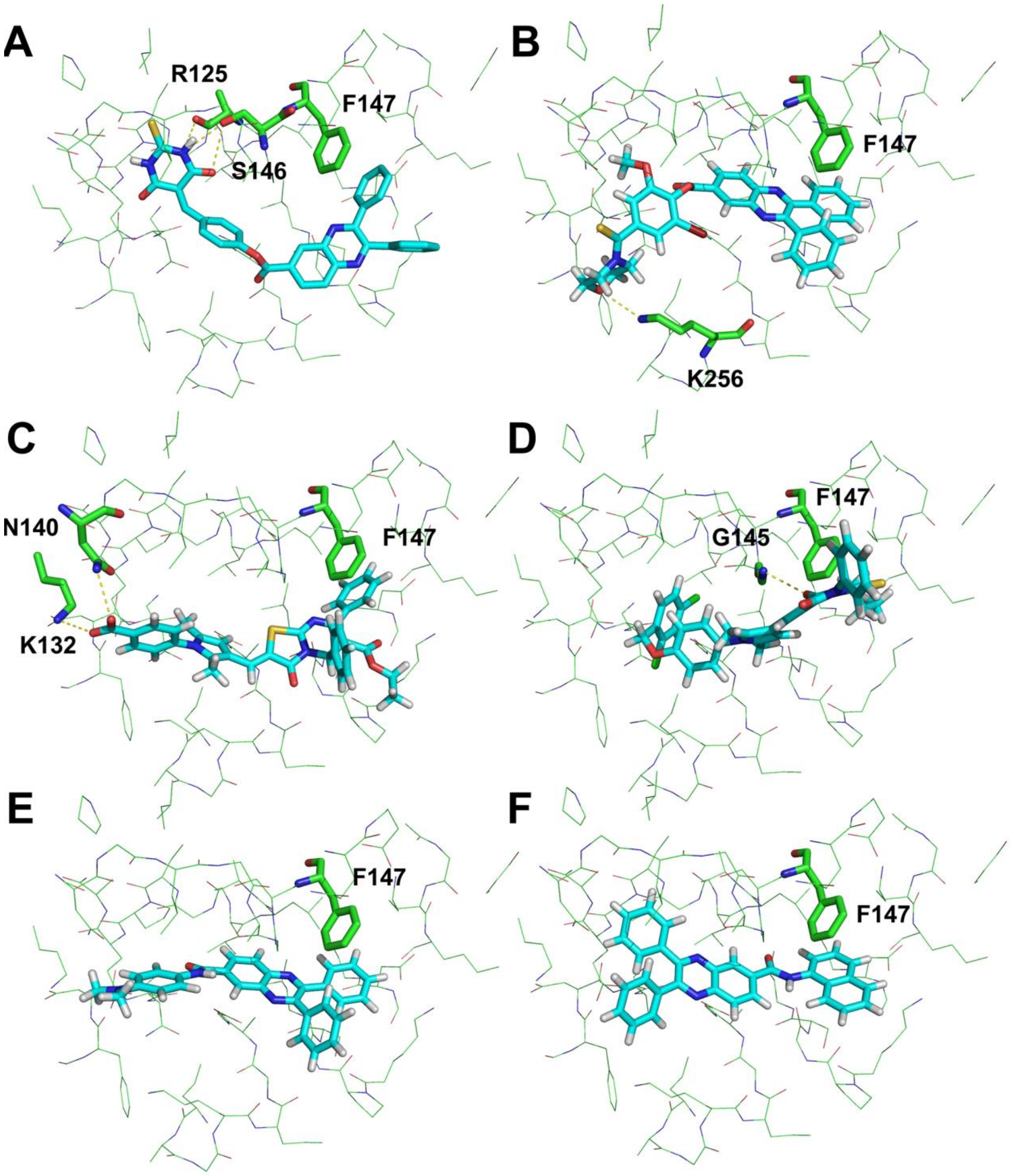

Figure S7. The binding mode of compound 1 (A) versus compounds 1-1 to 1-5 (B, C, D, E, F). F147 and polar residues forming H-bonds were in green sticks. Compounds were shown in cyan sticks

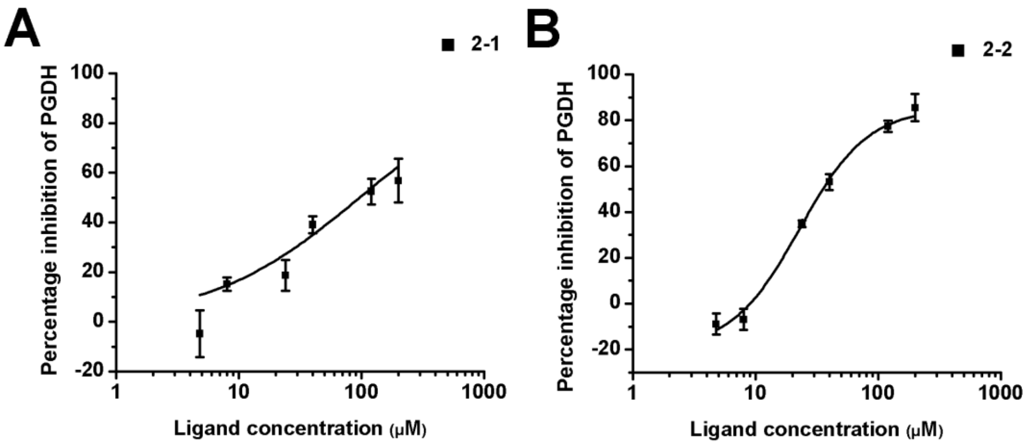

Figure S8. Kinetics of compounds 2-1 and 2-2. (A-B) Dose-response curves for compounds **2-1** and **2-2**. The IC_{50} values were 96 ± 22 for **2-1**, 22.3 ± 2.5 μM for **2-2**.

.

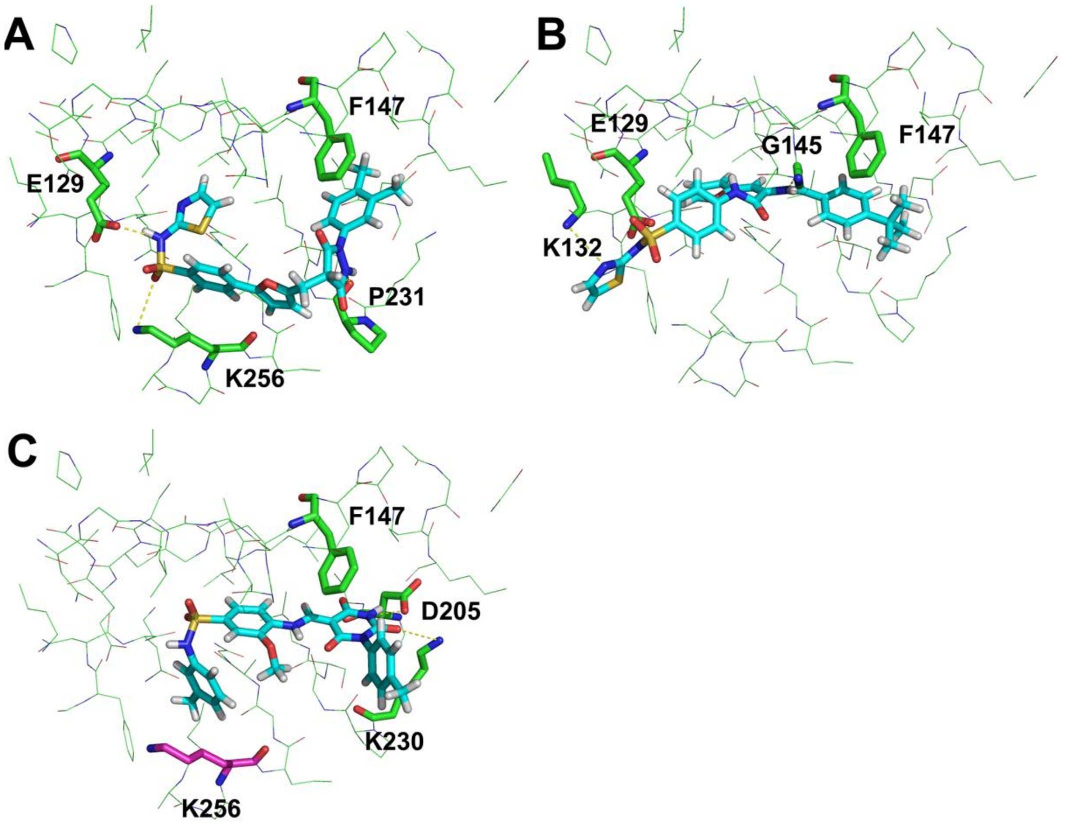

Figure S9. The binding mode of compound 2 and analogs with site I. Compounds **2** (A), **2-1** (B), and **2-2** (C) were in cyan sticks. F147 and polar residues forming H-bonds with compounds were in green sticks. K256 for cation-π interaction was shown in magentas stick.

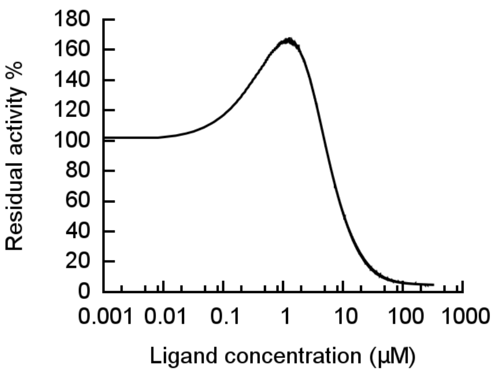

Figure S10. Computational simulation. Example simulation result of reaction Scheme S1 with parameters: K_1-K_{10} (μM): 0.390, 23.7, 0.00100, 0.0632, 11.2, 0.0207, 0.000718, 0.442, 0.00699, 1.16; k_1-k_4 (s^{-1}): 0.275, 7.57, 0.0113, 0.1826; initial concentration of the enzyme: 1.37 μM, substrate: 500

μM.

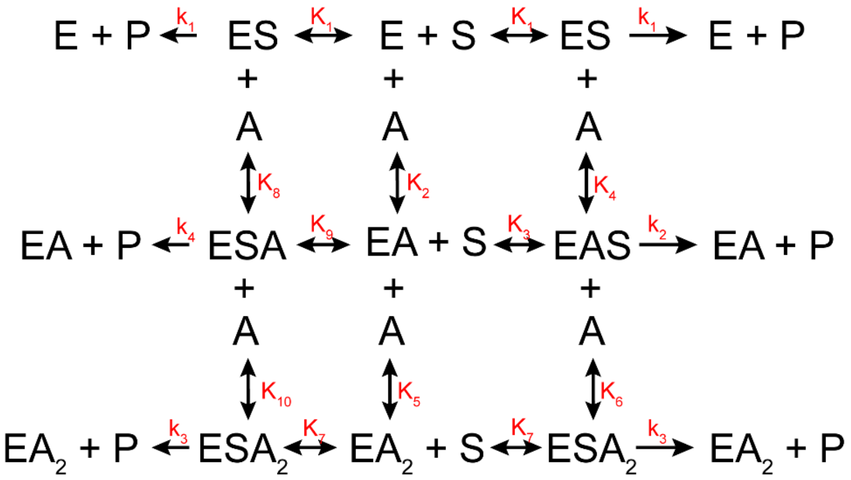

Scheme S1. Proposed reaction mechanism of PGDH dimer (E) with its substrate (S) and the effector molecule (A). In the triple complex EAS, S and A were bound to different subunits, whereas in ESA, they were bound to the same subunit.
